# Formation of Iron (Hydr)Oxide Nanoparticles with a pH-Clock

**DOI:** 10.3390/nano12213743

**Published:** 2022-10-25

**Authors:** Ronny Kürsteiner, Yong Ding, Maximilian Ritter, Guido Panzarasa

**Affiliations:** Wood Materials Science, Institute for Building Materials, ETH Zürich, Laura-Hezner-Weg 7, 8093 Zürich, Switzerland

**Keywords:** magnetite, lepidocrocite, pH, clock reaction, materials programming, systems chemistry, self-assembly, iron oxides, formaldehyde, sulfite

## Abstract

We demonstrate the autonomous synthesis of iron (hydr)oxide (green rust, magnetite, and lepidocrocite) nanoparticles by precipitating iron(II) ions using hydroxide ions generated in situ with the methylene glycol-sulfite (MGS) reaction, a pH-clock. We show that the nature of the products can be predetermined by tuning the initial iron(II) concentration.

## 1. Introduction

A huge variety of inorganic colloids and nanoparticles can be synthesized by pH-controlled precipitation reactions in aqueous solutions [1]. The required pH changes can be achieved either by adding acids or bases directly or by generating them in situ. The first approach needs external control and it can lead to unwanted high local concentrations of the precipitating agent, which in turn affects the size distribution and morphology of the resulting particles [2]. The use of light-triggered systems, such as photo-acids/-bases [3], or performing reactions under hydrothermal conditions (that is, at high temperature and pressure in specially designed vessels) [4] are common approaches for changing pH in situ. Nevertheless, they still rely on external stimuli and do not allow the programming of particle synthesis in the time domain.

Previous works demonstrated the usefulness of pH-driven clock reactions, chemical systems able to generate sudden pH changes after a tailorable time delay [5,6], for materials science applications [7] and especially for the time-controlled self-assembly of polymer-based [2,8] and supramolecular colloids [9,10], gels [11,12], and inorganic particles [13,14]. Recently, such an approach has been applied for the synthesis of a zinc-imidazolate metal–organic framework (ZIF-8) [15]. Here, we want to extend our method to fully inorganic particles, more precisely to iron-based ones.

It is known that, depending on the reaction conditions (especially pH and air exposure), the alkaline precipitation of iron(II) ions can give rise to a rich variety of hydroxides (e.g., “green rusts”), oxyde-hydroxides (e.g., lepidocrocite, goethite), and oxides (e.g., magnetite) [16,17]. We will show that the use of an alkali-generating clock, such as the methylene glycol-sulfite (MGS) reaction, allows the synthesis of a variety of iron (hydr)oxides (green rust, magnetite, and lepidocrocite) in an autonomous, programmable way.

The methylene glycol-sulfite (MGS) reaction, or “formaldehyde clock”, is a well-known acid-to-alkali clock reaction (ΔpH up to 5.5) [2,8,10,17,18]. In nuce, formaldehyde CH_2_O (from the spontaneous dehydration of methylene glycol MG, CH_2_(OH)_2_, Equation (1)) reacts rapidly with sulfite SO_3_^2−^, generating hydroxymethanesulfonate HOCH_2_SO_3_^−^ (HMS) and hydroxide ions OH^−^ (Equation (2)). The latter ions are immediately scavenged by bisulfite HSO_3_^−^, which transforms into sulfite (Equation (3)):

(1) $$CH_{2}{\left(OH\right)}_{2}⇋CH_{2}O+H_{2}O$$

(2) $$H_{2}O+CH_{2}O+SO_{3}^{2-}\to HOCH_{2}SO_{3}^{-}+OH^{-}$$

(3) $$HSO_{3}^{-}+OH^{-}\to SO_{3}^{2-}+H_{2}O$$

Since bisulfite is present in large excess, formaldehyde reacts much faster with sulfite than with bisulfite [18], and the pH remains fairly constant at ca. 5.5 until bisulfite is completely used up. Then, it suddenly rises to pH 10.5. The final maximum concentration of base which can be generated by the MGS clock corresponds directly to the initial sulfite concentration (ca. 5 mM for our conditions). For a constant sulfite/bisulfite ratio, the lagtime depends on the initial MG concentration. 

## 2. Results and Discussion

In our experiments, we fixed the initial concentrations of sulfite, bisulfite, and methylene glycol at 5 mM, 50 mM, and 200 mM, respectively. These conditions correspond to an average lag time of 25 s before the sudden pH increase. We chose iron(II) chloride FeCl_2_ as the iron source in concentrations ranging from 0.1 mM to 5 mM.

An intense yellow color developed immediately upon the addition of 1 mM Fe(II) to the sulfite–bisulfite solution [19]. The clock reaction was started by the addition of 200 mM MG. The presence of Fe(II) ions did not significantly change the initial lagtime, and an olive green flocculent precipitate was formed upon the sudden pH increase.

**Figure 1:**
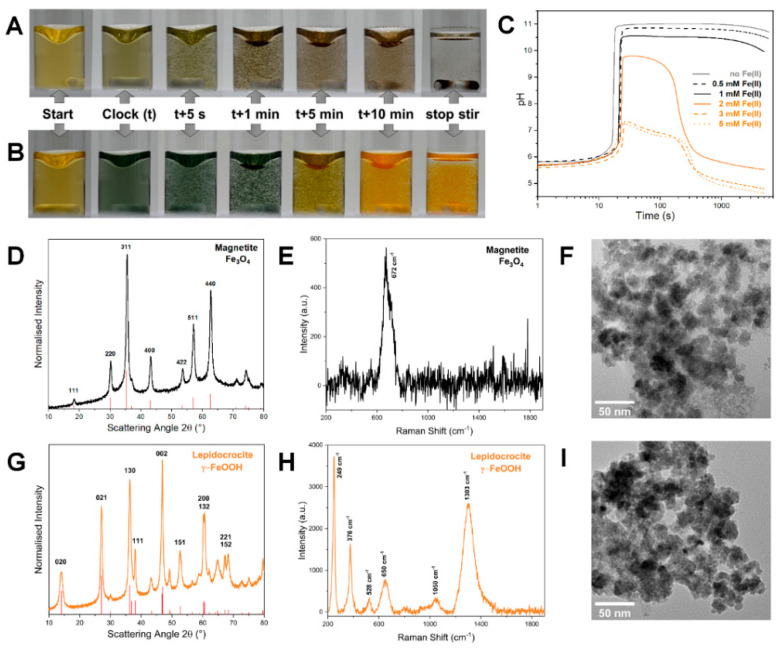
The MGS-Fe(II) system. (**A**,**B**) Photographic sequences showing the color evolution and precipitate formation for the MGS clock in presence of (**A**) 1 mM Fe(II) and (**B**) 2 mM Fe(II). In (**A**) particles are rapidly attracted by the magnetic stir bar once stirring is stopped, while in (**B**) the particles remain in suspension. (**C**) Evolution of pH over time for the MGS-Fe(II) system as a function of initial [Fe(II)]. From their XRPD diffractograms (**D**,**G**) and Raman spectra (**E**,**H**) the products have been identified as (**D**,**E**) magnetite and (**G**,**H**) lepidocrocite, respectively (the red lines represent the peaks of crystallographic standards). The TEM images (**F**,**I**) show the morphology of the products obtained with (**F**) 1 mM and (**I**) 2 mM Fe(II).

Within a few minutes, the precipitate turned dark brown-black and magnetic (Figure 1A, Video S1). However, when the Fe(II) concentration was increased to 2 mM, the initial precipitate was dark green and over time it changed into an orange, non-magnetic product (Figure 1B, Video S2). Moreover, this transformation was accompanied by a striking pH change (Figure 1C). The experiment was then repeated with different iron(II) concentrations. Decreasing the concentration of Fe(II) to 0.5 mM led to results identical to those obtained with 1 mM Fe(II). By contrast, with 3 mM Fe(II) the product was an orange precipitate, as for the 2 mM Fe(II) case, but the associated pH change was less intense. The same was observed when the Fe(II) concentration was increased to 5 mM.

The initial precipitate (dark green or olive, respectively, for 2 and 1 mM Fe(II)), is a “green rust” i.e., a mixed hydroxide of iron(II) and iron(III) containing additional structural anions (such as chloride and, possibly, HMS). Kept under stirring in contact with air, the precipitate evolved spontaneously within few minutes into different products, depending on the initial Fe(II) concentration.

By means of X-ray powder diffraction (XRPD), we were able to identify the dark brown magnetic product as magnetite Fe_3_O_4_ (Figure 1D) and the orange, non-magnetic one, as iron(III) oxyhydroxide γ-FeOOH i.e., lepidocrocite (Figure 1G). Both products are nanocrystalline with crystallite sizes (calculated from XRD data) of 12.5 ± 1.0 nm and 7.5 ± 1.1 nm for magnetite and lepidocrocite, respectively. The Fe_3_O_4_ lattice parameter (calculated from the fitted peak positions) of 8.385 Å indicates that the magnetite phase was Fe(II)-deficient [20], which can result in the partial formation of structurally related maghemite (γ-Fe_2_O_3_). Maghemite is often associated to magnetite, and their differentiation by means of XRD would require a detailed investigation which is outside the scope of this work [21]. Nevertheless, Raman spectroscopy (Figure 1E) confirmed the main phase to be magnetite [22]. The calculated lattice parameters for the lepidocrocite phase were $a_{0}=3.872\;Å,\;b_{0}=12.65\;Å,$ and $c_{0}=3.054\;Å$. While $a_{0}$ and $c_{0}$ corresponded to reported values [23], $b_{0}$ showed a slight deviation which can be attributed to the experimental uncertainty at small diffraction angles coupled with the strong fluorescence from the sample. As can be seen from its Raman spectrum (Figure 1H), the sample was confirmed to be lepidocrocite [22]. Transmission electron microscopy (TEM) images showed that both products consisted of the aggregates of nanoparticles (<20 nm in size) (Figure 1F (magnetite), Figure 1I (lepidocrocite)). Scanning transmission electron microscopy (STEM) (Figures S1 and S3) and energy-dispersive X-ray spectroscopy (EDS) (Figures S2 and S4) were used to perform the elemental analysis of both samples. The products were also characterized using Fourier-transform infrared spectroscopy (FTIR) [24,25,26] (Figure S5). The FTIR spectrum of the magnetite sample suggests that goethite could be present, although as a minor impurity.

In our MGS-Fe(II) system, magnetite always forms when [Fe(II)] ≤ 1 mM and the associated pH-time evolution is very similar to that of the MGS clock without Fe(II). On the other hand, for [Fe(II)] ≥ 2 mM, the final product is lepidocrocite and its formation is accompanied by a substantial pH decrease. It is known from the literature that when a mixture of iron(II) and iron(III) hydroxides is slowly oxidized (e.g., by exposure to atmospheric oxygen), the nature of the product(s) depends strongly on the pH of the reaction mixture. In general, the formation of magnetite is favored for pH ≥ 8, while for more acidic pH values (pH 5–7) lepidocrocite is formed [1,15]. The decrease in the maximum pH observed for increasing [Fe(II)] can be explained by the consumption of the base produced by the MGS clock to generate Fe(OH)_2_, based on a stoichiometry of two equivalents of hydroxide ions per equivalent of Fe(II). For [Fe(II)] ≥ 2 mM, a subsequent pH decrease accompanies the transformation of green rust into lepidocrocite, a behavior which could be accounted for by referring [15] to Equation (4):

(4) $$Fe_{y}^{II}Fe_{x}^{III}{\left(OH\right)}_{3x+2y-z}Cl_{z}+0.25yO_{2}+zOH^{-}\to \;\left(x+y\right)FeOOH+zCl^{-}+\left(x+0.5y\right)H_{2}O$$

Performing the experiments under oxygen-free conditions (that is, under a nitrogen atmosphere and using deoxygenated solutions), the only product obtained was always green rust, regardless of the initial [Fe(II)] concentration, and the final pH remained constant. These results confirmed the role of atmospheric oxygen in the subsequent reactions and that the dynamics of the MGS clock reaction were not influenced by sulfite-induced Fe(II) oxidation [27].

In conclusion, we report the application of the methylene glycol-sulfite (MGS) reaction, an acid-to-alkali clock, for the time-controlled synthesis of iron (hydr)oxide nanoparticles. Our approach could be applied to other metal cations as well, if their inertness towards the MGS clock and limited solubility in water of the corresponding (hydr)oxide products are adequately addressed.

Our results further demonstrate the importance of chemical clocks in materials science and represent an important step for materials programming. Even though our approach is not as practical for the synthesis of iron (hydr)oxides as for more conventional types, it is nevertheless robust, reproducible, and up-scalable (from 10 mL to 1 L reaction volume). We are currently investigating its application to other transition metal cations and for the synthesis of layered double hydroxides.

## 3. Materials and Methods

*Reagents.* Sodium sulfite (≥98%), sodium bisulfite (ACS reagent, mixture of NaHSO_3_ and Na_2_S_2_O_5_), sodium hydroxymethanesulfonate (formaldehyde-sodium bisulfite adduct, HMSNa, 95%), and iron(II) chloride tetrahydrate FeCl_2_•4H_2_O (ReagentPlus^®^, 98%) were purchased from Sigma-Aldrich (Buchs, Switzerland). Concentrated aqueous formaldehyde solution (37–41%, stabilized with 12% methanol) was purchased from Fisher Scientific (UK). Sodium hydroxide NaOH aqueous solution (ConvoL NORMADOSE 1N) was purchased from VWR (Stříbrná Skalice, Czech Republic). Unless otherwise stated, all chemicals were of analytical or reagent grade purity and used as received. Water was purified using a MilliQ system (resistivity 18.2 MΩ·cm).

*Measurement of pH.* A Hanna Instruments (Woonsocket, RI, USA) HI5222-02 benchtop pH-meter was used together with a HI1330B glass body combination pH microelectrode from the same Company. The pH-meter was calibrated with standard buffer solutions (pH values: 1.67, 4.01, 7.01, 10.01, 12.45) before each set of analysis. The pH-electrode was cleaned after each analysis by repeated immersion in water, the excess water gently removed with hairless paper, and immediately immersed in the solution to analyze. The pH-meter was interfaced with a computer through the software HI92000–5.0.38 (Hanna Instruments, USA) to allow continuous recording of pH values with a time interval of 1 s.

*X-Ray powder diffraction (XRPD).* A PANalytical X’Pert PRO MPD diffracrometer using monochromated Cu K_α1_ radiation (40 kV, 45 mA) in Bragg–Brentano geometry was used. Samples for XRPD were synthesized on a 500 mL (lepidocrocite) or 1 L (magnetite) scale, washed twice with water and once with ethanol, and dried under vacuum at room temperature. The dried samples were ground using an agate pestle and mortar and deposited on a silicon low-background sample holder. The fluorescent background was suppressed by optimizing the lower pulse height distribution (PHD) limit. The best signal-to-noise ratio was obtained by changing the lower limit to 60%. The diffractograms were obtained from 5° to 80° with an acquisition time of 5 h and a step size of 0.0334°. Analyses were performed using HighScore Plus [28]. The crystallite size was determined using the Williamson–Hall method assuming a uniform deformation model. The corresponding reference cards [29] are PDF 04-009-2284 for magnetite and PDF 04-014-3986 for lepidocrocite.

*Transmission electron microscopy (TEM).* TEM was performed with a JEOL JEM 1400 instrument at a 120 kV acceleration voltage. To prepare the samples, particle suspensions in ethanol were deposited on continuous carbon-coated 400 mesh copper grids and air-dried.

*Scanning transmission electron microscopy (STEM).* Analytical STEM was performed on an FEI Talos F200X operated at 200 kV acceleration voltage. Elemental content distribution mapping was carried out by energy dispersive X-ray spectroscopy (EDS) STEM spectrum imaging with a windowless Bruker Super-X EDS system. The EDS spectra were acquired over the course of for 300 s up to 20 keV with a spectral resolution of 10 eV per channel. Quantification was performed using the Cliff–Lorimer method.

*Fourier-transform infrared spectroscopy (FTIR)*. FTIR in attenuated total reflectance (ATR) conditions were performed with a Bruker Alpha P spectrophotometer equipped with a diamond window. Spectra were acquired with a 4 cm^−1^ resolution.

*Raman spectroscopy*. Raman spectra were acquired with a confocal Renishaw inVia Raman microscope using a 30 W 532 nm laser, a 1800 lines mm^−1^ grating, and a Zeiss 20× objective. The exposure time was 20 s with 0.1% laser power and a total of 40 accumulations, covering a spectral range of 200–1900 cm^−1^.

*Experimental protocols.* A sulfite-bisulfite stock solution, containing 14 mM sulfite and 140 mM bisulfite, was prepared by dissolving 0.070 g of sodium sulfite and 0.58 g of sodium bisulfite in 40 mL of water. This solution was made fresh before use and discarded after two hours. A 6.5 M methylene glycol (MG) stock solution was prepared by diluting one part of the concentrated (ca. 13 M) aqueous solution with one part of water and aged overnight before use. For the control experiments, a 550 mM sodium hydroxymethanesulfonate (HMSNa) was prepared by dissolving 737.5 mg of HMS in 10 mL of water. A 50 mM iron(II) stock solution was prepared by dissolving 0.10 g FeCl_2_•4H_2_O in 10 mL of water. The iron(II) stock solution was prepared fresh before each experiment, filtered through a 0.22 µm poly(ether sulfone) syringe filter, and used within 1 h. All stock solutions were stored at room temperature avoiding direct light.

In a typical reaction, the reactants were mixed in the following order: water, metal salt solution, sulfite-bisulfite solution, and eventually the methylene glycol solution. After the reaction, the precipitates were collected by centrifugation, washed first with water then with ethanol (redispersion is aided with gentle sonication), and eventually dried under vacuum. Reactions were carried out at room temperature (23 ± 1 °C). Reactions under air on a 10 mL-scale were conducted in 15 mL-glass vials, mixing with PTFE-coated magnetic stirrer bars (5 × 20 mm) rotating at 500 rpm. Upscaled reactions (e.g., to obtain enough sample for XRPD) were conducted under air on a 500 mL scale (lepidocrocite, conducted in 1 L glass beakers, mixing with 80 mm PTFE-coated magnetic stirrer bars rotating at 350 rpm) or 1 L scale (magnetite, conducted in 2 L glass beakers, mixing with 80 mm PTFE-coated magnetic stirrer bars rotating at 405 rpm). Reactions under inert atmosphere (nitrogen) were carried out on 10 mL- and 100 mL-scales in, respectively, 20 mL and 250 mL Schlenk flasks, mixing with PTFE-coated magnetic stirrer bars rotating respectively at 700 rpm and 800 rpm. To measure the pH evolution over time for the MGS-Fe(II) system, the reactions were carried out in 250 mL tall beakers open to air with 100 mL (final volume) of the reaction mixture.

## Data Availability

Not applicable.

## Supplementary Materials

The following supporting information can be downloaded at: https://www.mdpi.com/article/10.3390/nano12213743/s1, Additional data (Figures S1–S5), Video S1: 1 mM Fe(II)_Magnetite. Video S2: 2 mM Fe(II)_Lepidocrocite.
