# Supplementary material for: Formation of Iron (Hydr)Oxide Nanoparticles with a pH-Clock
Source: Nanomaterials (Basel). 2022 Oct 25;12(21):3743. doi: 10.3390/nano12213743 (PMC9653707; doi:10.3390/nano12213743)
Supplement: Supplementary file 1 [file nanomaterials-12-03743-s001.zip › nanomaterials-1889520-supplementary.pdf]

SUPPORTING INFORMATION

Formation of Iron (Hydr)Oxide Nanoparticles with a pH-Clock

Ronny Kürsteiner, Yong Ding, Maximilian Ritter and Guido Panzarasa *

Wood Materials Science, Institute for Building Materials, ETH Zürich, Laura-Hezner-Weg 7, 8093 Zürich, Switzerland
* Correspondence: guidop@ethz.ch

Abstract: We demonstrate the autonomous synthesis of iron (hydr)oxide (green rust, magnetite, and lepidocrocite) nanoparticles by precipitating iron(II) ions using hydroxide ions generated in situ with the methylene glycol-sulfite (MGS) reaction, a pH-clock. We show that the nature of the products can be predetermined by tuning the initial iron(II) concentration.

Keywords: magnetite; lepidocrocite; pH; clock reaction; materials programming; systems chemistry; self-assembly; iron oxides; formaldehyde; sulfite

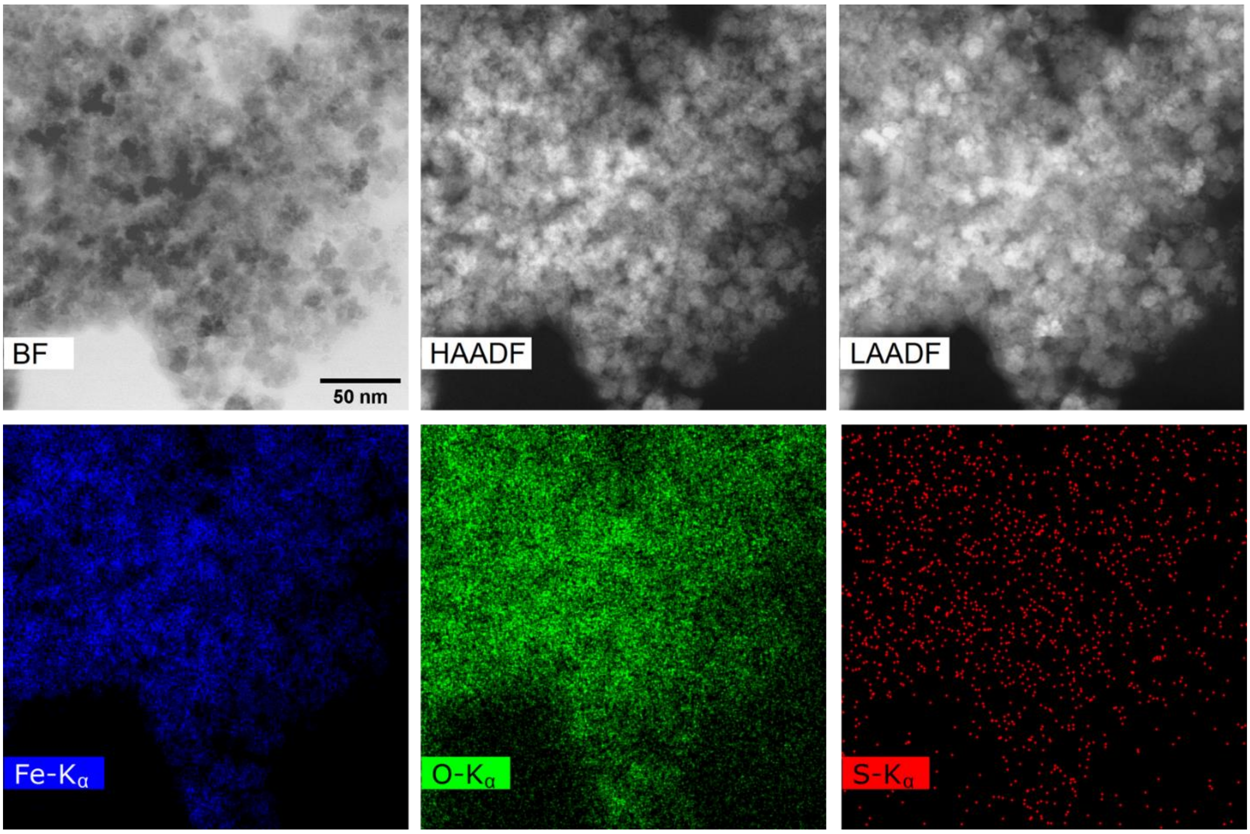

Figure S1. STEM images (bright field BF, high-angle annular dark field HAADF, low-angle annular dark field LAADF) and elemental maps showing the distribution of iron, oxygen and sulfur for a magnetite sample synthesized using the MGS clock.

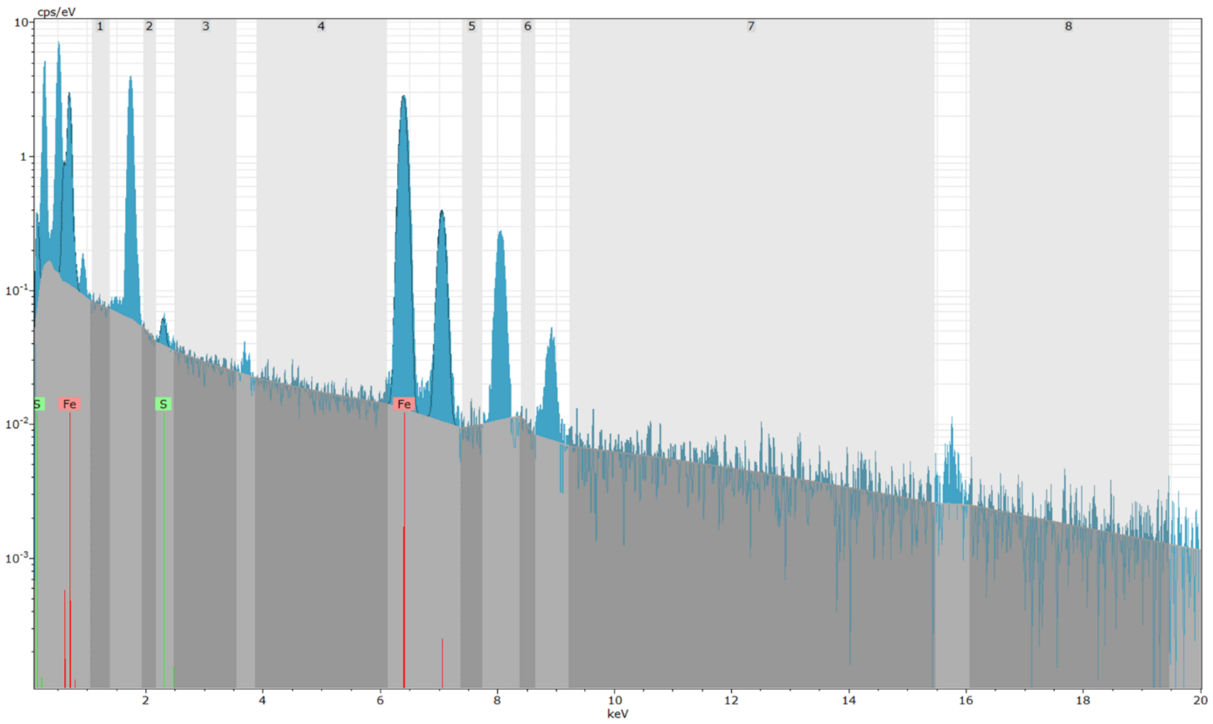

Element	*Content (wt%)*	*σ (wt%)*	*Content (at%)*
Iron (K-Series)	99.60	3.05	99.30
Sulfur (K-Series)	0.40	0.05	0.70

Figure S2. EDS quantification (Cliff-Lorimer method) of iron and sulfur in a magnetite sample synthesized using the MGS clock. Apart form the expected elements in the sample (Fe, O, S, C), and fluorescence artifacts from the instrument, detector, and sample holder (Si, Al, Cu, Zr), and build-up of contamination layer (C, O), a small amount of Ca (0.03 at% over-all) was detected. Since Ca is absent in the lepidocrocite sample, it is unlikely that it stems from precursor contamination.

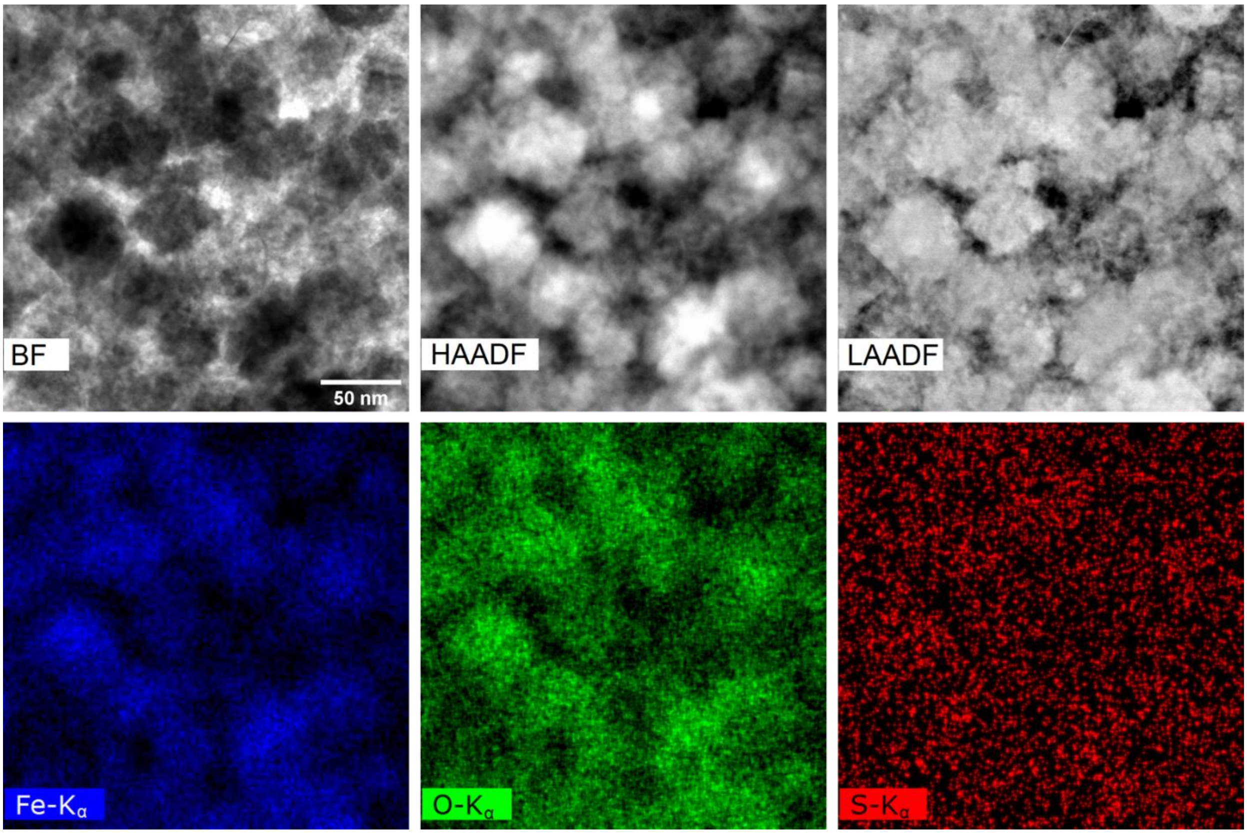

Figure S3. STEM images (bright field BF, high-angle annular dark field HAADF, low-angle annular dark field LAADF) and elemental maps showing the distribution of iron, oxygen and sulfur for a lepidocrocite sample synthesized using the MGS clock.

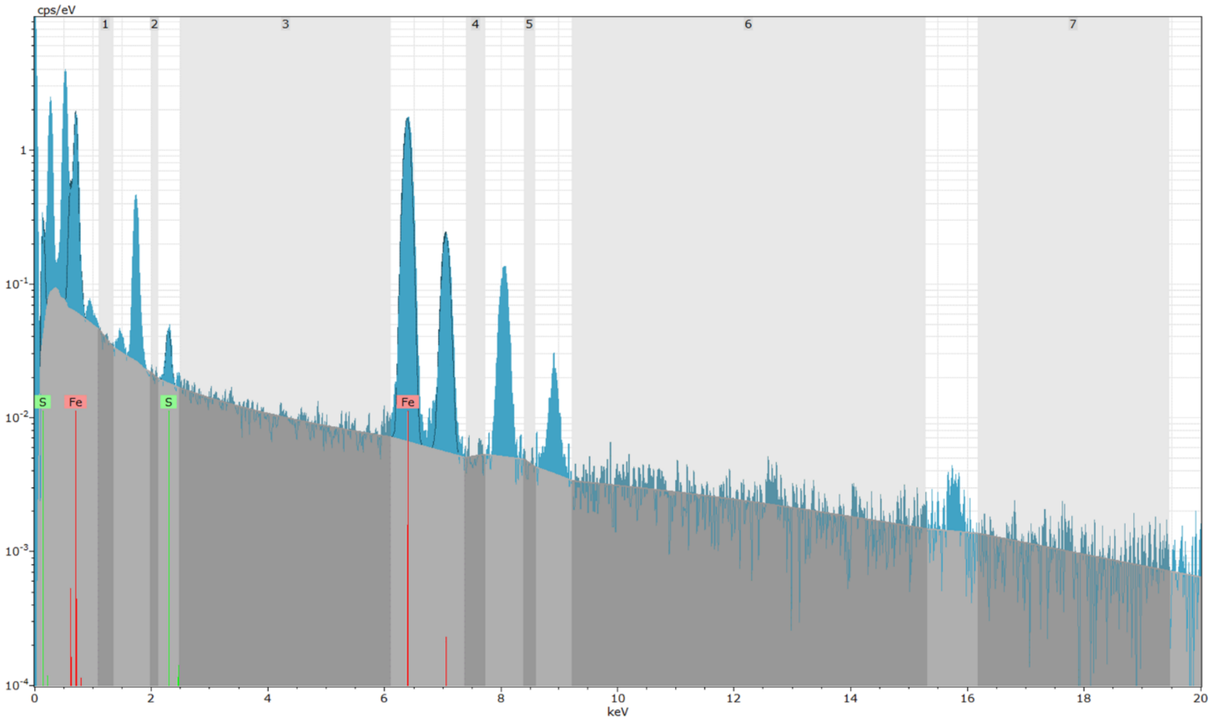

Element	*Content (wt%)*	*σ (wt%)*	*Content (at%)*
Iron (K-Series)	99.25	3.03	98.70
Sulfur (K-Series)	0.75	0.06	1.30

Figure S4. EDS quantification (Cliff-Lorimer method) of iron and sulfur in a lepidocrocite sample synthesized using the MGS clock. Apart form the expected elements in the sample (Fe, O, S, C), and fluorescence artifacts from the instrument, detector, and sample holder (Si, Al, Cu, Zr), and build-up of contamination layer (C, O), a slight Pb-Lβ_1 Peak (from radiation shielding of the column) is visible. Since the S-K series and the Pb-M series are in the same energy region, a quantification including Pb was performed. Taking Pb into account, the Fe and S contents are 98.8 at% and 1.11 at%, respectively.

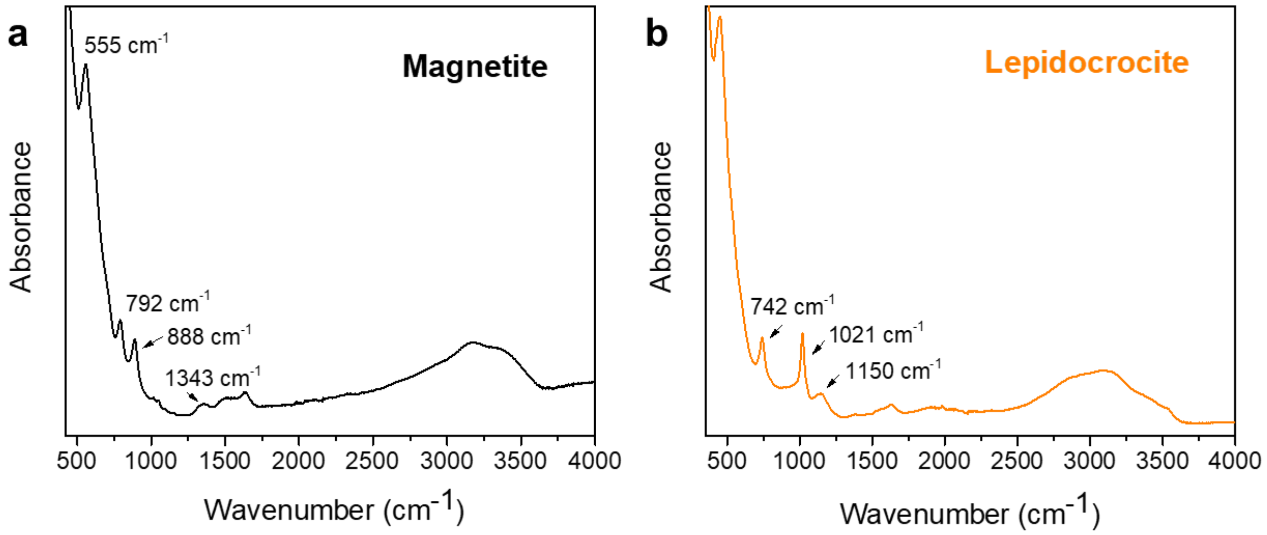

Figure S5. Normalized FTIR spectra of (**a**) magnetite and (**b**) lepidocrocite samples synthesized using the MGS clock. The spectra are in good agreement with literature ones [1,2]. In (**a**) the peak at 555 cm^{-1} can be assigned to the Fe–O bond vibration, while the peaks at 792 cm^{-1} and 888 cm^{-1} could be associated with Fe–OH vibrations from goethite [1]. The peak at 1343 cm^{-1} can be associated to the S=O stretching and thus to the presence of adsorbed hydroxymethanesulfonate. In (**b**) the peaks at 1150 cm^{-1}, 1021 cm^{-1} and 742 cm^{-1} are from vibrations that are characteristic of lepidocrocite [2]. Both magnetite and lepidocrocite samples show a broad absorption band from 2500 cm^{-1} to 3600 cm^{-1} associated to the stretching of C–H and –OH bonds.

References

[1] M. Stoia, R. Istratie, C. Păcurariu, Investigation of magnetite nanoparticles stability in air by thermal analysis and FTIR spectroscopy *J. Therm. Anal. Calorim.* **2016**, *125*, 1185–1198.

[2] D. G. Lewis, V. C. Farmer, Infrared absorption of surface hydroxyl groups and lattice vibrations in lepidocrocite (γ-FeOOH) and boehmite (γ-AIOOH) *Clay Min.* **1986**, *21*, 93–100.
